# Eighteenth Century Methods

**Published:** 1920-06-05

**Authors:** 


### THE VOLUNTARY HOSPITAL.

### Eighteenth Century Methods.

Lord Knutsford's signal of distress may save the London Hospital for the time being at all events. This is one of London's monuments, and large numbers of the citizens, rich and poor, will sacrifice much before they allow her doors to close.

During recent weeks I have been busily engaged helping forward a fête to keep open a Dublin hospital heavily encumbered with a war debt. This is Dublin's oldest hospital, built in the early part of the eighteenth century —hospital century—with the fortune of a famous physician and his sister, who sacrificed all that they possessed in order to provide Dublin's poor with skilled treatment in time of sickness. Doctor Steevens and his sister were the twin children of a Royalist clergyman who fled to Ireland as a refugee in the time of the Commonwealth because he had preached a sermon in defence of King Charles, and the Roundhead soldiers sought his blood. Swift and Stella were intimately associated with the hospital in its early days. And now this historic institution is crippled with a war debt of £10,000, the cost of playing the game at a time when the enemy was arranging for victory banquets in Paris and in London. I was one of a number of people voluntarily engaged in helping forward a fête to reduce this debt. For several weeks the work was constant and strenuous. The Honorary Organiser, one of Dublin's most devoted war-workers, toiled at the risk of her life, and against the urgent advice of her doctor. Ladies sat up all night making fantastic dresses for advertising men in the streets. Lorries with drumming parties paraded the thoroughfares announcing the fête. The hospital wore her glad rags and emptied every available space, indoors and out, to provide pleasure for citizens young and old. Hobby horses, swings, ballrooms, concert parties, flowers, country produce, bargains—everything to gladden the heart and please the eye.

And then what happened?

The tragedy and the misery of it.

It rained, and rained, and rained! The windows of heaven opened and down came the deluge, and of course the deluge won. In the middle of it all I saw Lord Knutsford's appeal in *The Times*.

And I have been wondering if we are facing this hospital breakdown in a proper manner. Is it just and reasonable—is it creditable—that the nation's unpaid war debts should be thrown on the shoulders of a few philanthropic and high-minded men and women, and that they should have to tickle the palates of pleasure-seekers in order to provide the sick poor with medical treatment and nursing? In practice it works out that the voluntary hospital depends for its life on the whimsicalities of the weather and of the pleasure-seeking public. The heroic efforts of the philanthropic are only one factor in the case.

Looking backward at some of the dark days of 1917 and 1918 I sometimes wonder what would have happened if the hospitals of these countries closed down. What a wet blanket for the fighters at the home front, and what a tonic for the enemy! "England's Hospitals Closed" would have been "wirelessed" round the Fatherland as a joy signal. The signal that went round England was "carry on."

Carry on! Carry on!
Things never were looming so black;
But show that you haven't a cowardly streak,
And though you're unlucky you never are weak.
Carry on! Carry on!

The hospitals carried on, with one only object, and for one only cause, the nation's weal. And now, apparently, it is only by appeals and blandishments and allurements and greater sacrifice that the war debts can be discharged.

I am not disposed to cry shame, because I am not convinced that other and more worthy methods have been tried. I do not think that the cause of the voluntary hospital has been effectually put before the country. The poor man, the middle-class man, and the rich owe an unpayable debt to the voluntary hospital, although the common belief is that its benefits are conferred only on the destitute. It is not in the course of their private practice that physician, surgeon, and nurse acquire their skill, but in the hospital wards. A professional man may have ten talents, but without such experience as a public hospital affords it would be impossible for him to excel. Not one in a thousand of the public gives this matter a thought. It has never flashed across their minds that the hospital for the sick which is in their midst is providing untold benefit for the whole community. If it did Lord Knutsford would not find it necessary to send up S.O.S. signals for the "London." Nurses would not have to clamour for reasonable conditions of service. Philanthropic men and women would not be driven to running popular amusements for the million.

We began in this manner, and we have so continued. I turned up an old record the other day, of 1700 or thereabouts, and found there an account of a certain doctor who was running a lottery and some comedy performances for the upkeep of a lying-in hospital. We still adhere to eighteenth-century methods. The public have not been trained to give. It took quite three years of war to train them to give to the Red Cross. What I suggest is required is a vigorous and thorough system of propaganda extending to the shop, the school, the office, the church. The Press would lend its powerful aid. The advantages of the voluntary system should be demonstrated, and the scientific effort made to ascertain what each class of the community ought to contribute per head, so as to arrive at uniformity of effort.

The public are largely ignorant, largely thoughtless, largely apathetic. But they are also largely honest and honourable, and if the facts are fairly presented my conviction is that the response will be generous.

